# Children’s Transitions to Adulthood and Midlife Parents’ Depressive Symptoms and Activities of Daily Living Conditions in the United States

**DOI:** 10.3390/ijerph20126151

**Published:** 2023-06-16

**Authors:** Xing Zhang, Anna M. Hammersmith

**Affiliations:** 1 College of Health Solutions, Arizona State University, Phoenix, AZ 85004, USA; 2 Department of Sociology, Grand Valley State University, Allendale, MI 49401, USA; hammeran@gvsu.edu

**Keywords:** transitions to adulthood, intergenerational relations, depressive symptoms, physical health

## Abstract

Background: Parents and children are close over the life course. However, these relationships often change as parents age and children enter adulthood. Today, the entrance into adulthood for children has become delayed and increasingly unstable. Such changes may interrupt the child’s acquisition of resources used to support themselves and their midlife parents, having implications for parents’ mental and physical health. The purpose of this study is to examine the role of adult children’s transitions to adulthood on parents’ mental and physical health. Methods: Using data from the National Longitudinal Study of Adolescent to Adult Health (Add Health) and the Add Health Parent Study (AHPS), we investigated how certain transitions to adulthood (e.g., education, marriage, residential independence, employment, parenthood, and incarceration) for children were linked to the mental and physical health of their midlife parents. Results: In sum, we found that children’s educational attainment was linked to fewer activities of daily living (ADL) limitations and depressive symptoms among parents. Children’s marriage and employment were also associated with fewer ADL limitations among parents. Conclusions: Our findings reveal that adult children’s circumstances are associated with the mental and physical health of their midlife parents.

## 1. Introduction

The relationship between parents and their children is one of the most significant and meaningful bonds that spans across the life course. The lives of parents and children are intertwined such that events in the lives of adult children likely have implications for the health and well-being of their midlife parents and vice versa [1]. In most parent–child relationships, parents provide children with instrumental and emotional support on the road to adulthood [2]. Then, as children become adults, they often acquire statuses associated with adulthood, including residential independence, completing education, attaining full-time employment, marriage, and parenthood [3,4]. These statuses are often accompanied by increased independence of adult children from their parents and may allow children to provide more instrumental and emotional assistance to their parents [5].

In recent decades, however, the pathways young people take to reach adulthood have become more varied, which may put the health of their parents at risk [6,7,8]. Today, many adult children live with their parents for longer, delay marriage and parenthood, and spend more years pursuing education than in the past [8,9]. At the same time, a number of children encounter obstacles on the road to adulthood, such as returning to the parental home, lack or loss of employment, singlehood or marital dissolution, or incarceration [3,4]. Delayed entrance into transitions to adulthood may mean that midlife parents will offer children instrumental and emotional assistance further into their adult years [10,11]. Likewise, adult children may have fewer resources to offer their parents. When children do not acquire certain markers of adulthood, parents may also worry about how their children will fare in the future [4,12]. Continued support and feelings of concern directed toward adult children likely have implications for the mental and physical health of parents as they age. Today, children’s entrance into adulthood has changed dramatically. Given the persistence of the parent–child tie over the life course, it is likely that changes in the status attainments of children and accompanying stress are linked to parental health. In particular, this study focuses on how adult children’s status attainment shapes parents’ mental and physical health, operationalized as depressive symptoms and ADL limitations, respectively. Incorporating indicators of both mental and physical health can increase our understanding of how children’s acquisition of various transitions to adulthood may relate to different dimensions of parental well-being.

Using data from Waves I–V of the National Longitudinal Study of Adolescent to Adult Health (Add Health) and Wave II of the Add Health Parent Study (AHPS), we investigate two central research questions. First, how are certain transitions to adulthood (e.g., education, relationship status, living arrangements, employment, parenthood, and incarceration) acquired by children associated with parents’ mental health (e.g., depressive symptoms)? Second, how are transitions to adulthood acquired by children associated with parents’ physical health (e.g., ADL limitations)? This study advances current research on adult children’s status attainment and parental health in several key ways. Many prior studies relied on cross-sectional data, which does not allow for the examination of parent–child relationships over time. Using nationally representative data, we track children’s status acquisitions throughout their journey into adulthood. Prior studies have also been limited by data that include only reports given by adult children or their midlife parents. We advance this body of work by examining adult children’s reports of their achievement of transitions to adulthood in conjunction with their parents’ reports of their own mental and physical health. Finally, our study takes a more holistic view of the pathway into adulthood by accounting for several unique markers of adulthood. As children become adults, they may acquire a number of different statuses within the same time period. Thus, it is important to account for the complexity of the transition into adulthood.

### 1.1. Life Course Perspective

Relationships between parents and their children are one of the most enduring ties experienced throughout the life course [13]. Despite the persistence of the parent–child relationship over time, this bond changes throughout the life course [5]. The life course perspective sheds light on how the lives of parents and children are intertwined with one another, also understood as linked lives [1]. Parents provide both instrumental and emotional support to their children during their younger years all the way through adolescence and well into young adulthood [14]. However, as both age, it is often expected that parents will provide less assistance to their children. In fact, exchanges of support between parents and children often reverse, with adult children providing increasing amounts of support to their midlife parents [5].

Age-related reversal of reciprocity has been challenged in recent decades as a result of changes in the transition to adulthood [11]. Expectations surrounding the timing and ordering of children’s achievement of what are commonly considered to be transitions to adulthood have shifted dramatically, such that it often takes much longer to achieve transitions to adulthood than in the past [6,7,8]. As of 2018, when the Wave V Add Health data were collected, about 1/3 of adult children aged 18 to 34 lived in a parent’s home relative to about 20% in the 1970s [7,15]. Age at first marriage and first birth have also steadily increased. The median age at first marriage for women today is nearly 28 years compared to about 22 years four decades ago. Similarly, age at first birth has risen from about 22 years of age to around 27 in a span of forty years [16]. Moreover, adult children often spend more time in higher education today than in the past [17]. Despite investments in higher education, it also takes young people longer to secure stable employment and become economically independent today than in decades prior [7,17,18]. Given such delays, adult children may take longer to achieve self-sufficiency than in the past [19].

The ramifications of delays in attainment of transitions to adulthood extend beyond children and into the lives of midlife parents. Parents and children may share stress as a result of occurrences in one or the other’s lives. This is referred to as stress contagion, or the transfer of stress across individuals who share a close bond [4,20,21]. Stress can be harmful for both mental and physical health. For instance, stress can be linked to mental health problems such as depression that develop more quickly in response to adverse events [22]. Stress may also slowly erode health over time through a process known as weathering. Thus, physical health conditions that develop over a longer period of time—such as ADL limitations—may tie into stress [23]. There are two key mechanisms through which stressors in an adult child’s life may spill over into the lives of their midlife parents. First, parents may feel a continued sense of obligation to financially, instrumentally, and emotionally support children on their pathway into adulthood. Second, parents may also feel a sense of worry or concern for their children if they encounter challenges on the pathway to adulthood [4].

Children’s attainment of transitions to adulthood is often associated with achieving self-sufficiency through the accumulation of valuable resources, which can benefit not only the adult child, but also their midlife parents. Children who become more self-sufficient often require fewer resources from their parents [11]. Further, prior work indicates that adult children with greater resources are more likely to provide assistance to their midlife parents than their counterparts with fewer available resources [24]. In recent years, parents often act as a safety net of instrumental and social support for their children for longer, even if the parent has limited resources to share [6,7,10,11]. Similarly, delayed acquisition of transitions to adulthood also suggests that children will have fewer resources to offer their midlife parents. Parents’ continued support of adult children may be emotionally, economically, and socially taxing, and could potentially harm midlife parents’ health [4,25]. Thus, having children with delayed attainment of transitions to adulthood may leave older parents more vulnerable when it comes to protecting their own health as they age.

Parents also have a vested interest in their children’s entrance into transitions to adulthood. Parents are invested in their children and when children have difficulty acquiring statuses that are symbolic of adulthood, parents may grow concerned [26]. More specifically, parents may worry about how their children will fare in the future if they are unable to achieve self-sufficiency and independence through entrance into adult roles [4]. Feelings of worry or concern may link to increased physiological and psychological stress, ultimately leading to poorer mental and physical health outcomes among midlife parents [4,12].

### 1.2. Children’s Attainment of Transitions to Adulthood and Parental Health

Prior studies show how the acquisition of various transitions to adulthood may link to parental health and well-being. Young adults today spend an increasing number of years pursuing higher education. On the one hand, while completing their degree, an adult child’s parents may provide them with additional financial, emotional, and instrumental support [27,28], which could negatively link to parental health. On the other hand, an adult child’s completion of higher education can indicate the child’s self-sufficiency and may also reduce parental worry. This may reduce stress, thereby protecting the midlife parents’ mental and physical health. There is a growing body of research supporting the positive relationship between children’s education and parents’ mental as well as physical health [29,30,31,32]. Prior studies suggest this association is a function of resources, such as increased health information and support flowing from children to parents, as well as parents’ positive perceptions of more highly educated children [29,30].

Children’s marital status may also be tied to parental health. Marriage can be indicative of a child’s independence. Relatedly, spouses tend to pool their economic and social resources [33,34], and thus, children may rely on parents less for assistance, thereby benefiting the parents’ mental and physical health. In fact, Kalmijn and de Graaf [35] found that parents who had children enter a marriage reported fewer depressive symptoms. While a child’s union formation appears to be beneficial for the parents’ health, the dissolution of a romantic partnership may be deleterious. If a child experiences a break up or divorce, this is often accompanied by resource loss and a need for additional emotional support [35], which may strain parental health. Indeed, several studies have found that parents with divorced children tend to report greater depressive symptoms [35,36,37]. To our knowledge, no studies have examined how adult children’s marriage or divorce may link to parents’ physical health. Marriage could relate to better physical health for parents since marriage may indicate that children need fewer resources from their parents. In contrast, an adult child’s divorce may link to poorer physical health for parents since divorce may increase children’s reliance on parents and contribute to the parents’ feelings of worry about the child’s well-being.

Along the pathway into adulthood, children often experience changes in residence. This might mean moving back to the parental home for some children or moving out to an independent residence for others. Changes in residence for children might signal other concurrent status transitions [38]. For instance, exiting the parental home may align with a marriage or new employment, whereas a move back to the parental home may indicate job loss or the dissolution of a romantic relationship. Thus, residential independence is likely accompanied by the acquisition of additional resources for adult children, thereby unburdening parents. In contrast, having a child move back into the parental home likely indicates a child’s dependence and need for support from their parent(s) [39,40]. This need for additional support from parents is associated with poorer mental health for midlife parents when an adult child returns home [38]. Although children’s residential independence has a relationship with midlife parents’ mental health, it is also likely that this extends to physical health. An adult child’s move out of the parental home could improve physical health for parents as parents may need to provide children with fewer resources. However, when an adult child moves back into the parental home, this could elevate parental worry and also indicate a child’s need for parental support, which could be associated with poorer physical health.

Children’s job loss or gain may also be associated with parental health. When a child loses a job, it may indicate the loss of resources such as income or health benefits. Thus, children often become more reliant on parents for assistance after losing employment [11]. A child’s job loss can also spark concern among parents, which can threaten their health and well-being [41]. In contrast, entering employment likely increases children’s available resources that can used to support themselves as well as their midlife parents, which is likely associated with better health for parents. Thus, although no studies to date have found a link between adult children’s employment and midlife parents’ mental or physical health, it is likely that entering employment relates to better health through increasing children’s independence and reducing parental concern about child well-being. However, children’s job loss may indicate the opposite. A child’s loss of employment may increase their reliance on parents and contribute to parental concern about the child’s future.

When a child becomes a parent, there is evidence that this major life event may also be associated with changes in health and well-being for a midlife parent. Entrance into parenthood can often bring a lot of joy not only for the child, but also for the parent [42]. Despite this, some studies have revealed that parental support of children often increases when an adult child enters parenthood [33,43]. However, it is possible that parental support of children entering parenthood may be largely contingent on children’s other characteristics, such as age and partnership status [33,44]. Thus, it is unclear whether an adult child’s entrance into parenthood requires more emotional and instrumental assistance from midlife parents relative to childless children. Given this conflicting evidence, it is difficult to predict how a child’s entrance into parenthood may be tied to mental and physical health for parents.

A child’s incarceration can also play a critical role in shaping parental health. An adult child’s incarceration is a stressful experience not only for the child, but also for their older parents [45]. Further, parents may have to step in to provide additional emotional and financial resources for an incarcerated child. This may spill over into parental mental and physical health. In fact, research has indicated maternal health—both mental and physical—suffer following a child’s incarceration [45].

### 1.3. Other Factors Related to Children’s Status Acquisition and Parental Health

When considering the relationship between the children’s attainment of transitions to adulthood and parental health, it is crucial to account for several potential confounding factors. First, we account for parents’ gender. Aging mothers tend to be more invested in children and report closer ties with offspring than aging fathers [46]. This suggests that mothers’ health may be more strongly linked to children’s acquisition of transitions to adulthood than fathers’. We also control for parents’ race and ethnicity. Family closeness is often higher among Black, Latinx, and Asian families in the United States. Families of color often exchange more support with one another than White families [47]. Although exchanges of support are more common in non-White families relative to White families, families of color often have fewer resources to share [47]. Moreover, children in families of color often face more challenges when acquiring transitions to adulthood than children of White families [4]. Limited resources and challenges on the road to adulthood may put families of color at a greater risk of poor health outcomes relative to their White counterparts.

In addition to race and ethnicity, we account for immigrant status. Solidarity between family members in immigrant families is typically quite high [48], leading to more frequent exchanges of support between family members. Immigrant status may also shape norms around what are commonly considered to be transitions to adulthood. For instance, it is often considered normative to live with parents further into adulthood among some immigrant groups [7]. Greater family solidarity among immigrant families serves as a way to protect cultural values and norms, but is also way to help group members get ahead, as some immigrants face unique challenges on the road to adulthood [49].

We also control for the parents’ age. Older parents will likely have more health issues—especially physical health limitations—than their younger counterparts. Finally, we account for the parents’ education as a proxy for socioeconomic status. Parents with more socioeconomic resources will likely be better able to devote resources toward their own health and provide for adult children on the pathway to adulthood [25,43], suggesting that parents with higher education will benefit from better health compared to their peers with fewer years of education.

### 1.4. Hypotheses

We expect the following:

1. Parents of children who experience the acquisition of transitions to adulthood associated with greater resources (higher levels of educational attainment, marriage, parenthood, living independently, being employed, and not being arrested) by their late thirties will have better overall health—that is, fewer depressive symptoms and ADL conditions.
2. Parents with children who experience delayed acquisition of transitions to adulthood linked to greater resources or setbacks on the road to adulthood (lower levels of educational attainment, not being married or being divorced, never experiencing parenthood, living with parents, being unemployed, and experiencing arrest) by their late thirties will have poorer overall health—specifically, higher depressive symptoms and greater ADL conditions.

## 2. Materials and Methods

To examine our research questions, we use data from Wave V of the National Longitudinal Study of Adolescent to Adult Health (Add Health), and Wave II of the Add Health Parent Study based in the United States from the University of North Carolina at Chapel Hill. Add Health is a school-based, nationally representative sample of 20,745 adolescents in the 7–12th grades from 1994 to 1995 at Wave I. From Wave I, adolescents were followed up at Wave II from 1995–1996, Wave III from 2001–2002, Wave IV from 2008–2009, and Wave V from 2016–2018. The Add Health Parent Study interviewed 2013 parents of the Add Health children from 1994–1995 at Wave I and from 2016–2018 at Wave II. We link the data for adult children who completed Wave V of Add Health and had a parent who completed Wave II of the Add Health Parent Study, leading to a sample size of 1548 adult children and their parents.

### 2.1. Independent Variables

#### Transitions to Adulthood

Based on prior studies examining adult status changes [4], we examine the following six statuses commonly associated with adulthood at Wave V: educational attainment, romantic relationship status, living arrangements, employment status, parenthood status, and whether the respondent had ever been incarcerated. The respondents’ highest educational attainment included the following categories: less than high school (1), high school or GED (2), some college (3), completed college (4), and completed more than a college degree (5). The respondents’ romantic relationship status included whether the respondent was married (1), separated, divorced, or widowed (2), or never married (3). The respondents’ residence included whether the respondent lived on their own (1), lived with their parents (2), or lived in another living arrangement (3). The respondents’ employment and parenthood status included whether they were unemployed (0) or employed (1) and were never a parent (0) or a parent (1). Finally, we included a measure of whether the respondent had ever been arrested (1) or not (0).

### 2.2. Controls

We controlled for the parents’ gender, race and ethnicity, age, immigrant generation status, and educational attainment based on information from Wave II of the Add Health Parent Study. Gender included whether the respondent identified as a man (1) or woman (2). Race and ethnicity included the categories of whether the parent identified as non-Latinx white (1), non-Latinx Black (2), Latinx (3), or non-Latinx Asian (4). Immigrant generation status included whether the parent was born in the United States (0) or born outside of the United States (1). The parents’ highest educational attainment included the following categories: less than high school (1), high school or GED (2), some college (3), completed college (4), and more than a college degree (5).

### 2.3. Dependent Variables: Depressive Symptoms and ADL Conditions

#### 2.3.1. Depressive Symptoms

We included a measure of depressive symptoms based on the Center for Epidemiologic Studies—Depression Scale [50] from the following questions asked of the Add Health parents from Wave II of the Add Health Parent Study: “How often was each of the following things true during the past seven days?” Items included feeling unable to shake off the blues, even with help from family and friends, feeling depressed, feeling happy (which was reverse-coded), feeling sad, and feeling that life was not worth living (Cronbach’s alpha = 0.79). Responses ranged from never or rarely (1), sometimes (2), a lot of the time (3), to most of the time or all of the time (4).

#### 2.3.2. Activities of Daily Living (ADL) Conditions

The sum of ADL conditions was based on the following yes-no questions asked of parents from Wave II of the Add Health Parent Study: “Because of a health or memory problem, do you have any difficulty with bathing or showering?” The same question was asked for parents who had difficulties dressing and putting on shoes and socks, walking across a room, and difficulty getting up from a chair after sitting for long periods (Cronbach’s alpha = 0.64). Responses ranged from experiencing no ADL conditions (0), experiencing one ADL condition (1), experiencing two ADL conditions (2), experiencing three ADL conditions (3), to experiencing all ADL conditions (4).

### 2.4. Analytic Strategy

To conduct our research analyses, we first generated summary statistics of the respondents’ and parents’ summary statistics. Second, as the outcomes were continuous measures, we ran OLS regressions to examine associations between statuses acquired by adult children (educational attainment, romantic relationship status, residence, employment status, parenthood, and incarceration) and their parents’ depressive symptoms and ADL conditions. In order to examine the association of each adult status and parental health, we ran regressions individually for each of the six statuses acquired by adult children and included control variables. In order to examine the relative importance of each adult status relative to others, we ran regressions including all six transitions to adulthood along with control variables. All analyses included sample weights to ensure that the results were nationally representative.

## 3. Results

### 3.1. Table 1 Summary Statistics for the Adult Children

Summary statistics for the adult children in the sample from Wave V of Add Health are shown in Table 1. Slightly over half of the sample (55%) were women. A majority of the sample was non-Latinx white (76%), followed by non-Latinx Black (12%), Latinx (9%), and non-Latinx Asian (4%). On average, adult children were about 38 years old although the sample included some as young as 33 or as old as 42. A majority of adult children were born in the United States (97%) and completed some college or more (84%). Over half of the adult children were married (61%), resided independently (88%), were employed (85%), and had a child (69%). Approximately 31% of the adult children had ever been arrested by their late thirties.

**Table 1:** Summary statistics of the adult children from Wave V Add Health.

Variable	Mean	SE	Range
Gender (woman)	0.55	0.01	1 to 2
Race and ethnicity			1 to 4
Non-Latinx white	0.76	0.01	
Non-Latinx Black	0.12	0.01	
Latinx	0.09	0.01	
Non-Latinx Asian	0.04	0.01	
Age of respondent	37.58	0.06	33–42
Immigrant generation status			1 to 2
1st generation	0.03	0.01	
2nd + generation	0.97	0.01	
Highest educational attainment by Wave V			1 to 5
Less than high school	0.04	0.01	
High school/General Educational Development	0.13	0.01	
Some college	0.42	0.01	
Completed college	0.22	0.01	
More than college	0.20	0.01	
Relationship status in Wave V			1 to 3
Married	0.61	0.01	
Widowed/divorced/separated	0.14	0.01	
Never married	0.24	0.01	
Living arrangement in Wave V			1 to 3
Your own place	0.88	0.01	
Parents’ home	0.07	0.01	
Other	0.05	0.01	
Employed in Wave V	0.85	0.01	
Ever had a child by Wave V	0.69	0.01	0 to 1
Ever arrested by Wave V	0.31	0.01	0 to 1
N	1548		

### 3.2. Table 2 Summary Statistics for the Adult Parents

Table 2 shows the summary statistics for the adult parents of the Add Health Parent Study at Wave II. A majority of the sample (97%) were women and non-Latinx white (78%). Parents were approximately 63 years old on average, and 94% were born in the United States. A majority of the parents had completed at least a high school degree (91%). On average, parents experienced at least 1.41 depressive symptoms in the preceding week, and experienced 0.38 ADL conditions.

**Table 2:** Summary statistics of Add Health Parents at Wave II of the Add Health Parent Study.

Variable	Mean	SE	Range	Alpha
Gender (woman)	0.97	0.00	1 to 2	
Race and ethnicity			1 to 4	
Non-Latinx white	0.78	0.01		
Non-Latinx Black	0.11	0.01		
Latinx	0.08	0.01		
Non-Latinx Asian	0.03	0.01		
Age of respondent	62.68	0.16	47 to 80	
Immigrant generation status			0 to 1	
1st generation	0.06	0.01		
2nd + generation	0.94	0.01		
Highest educational attainment by Wave V			1 to 5	
Less than high school	0.09	0.01		
High school/General Education Development	0.30	0.01		
Some college	0.34	0.01		
Completed college	0.16	0.01		
More than college	0.11	0.01		
Center for Epidemiologic Studies Depression Scale at Wave V	1.41	0.01	1 to 4	0.79
Sum of Activities of Daily Living conditions	0.38	0.02	0 to 4	
N	1548			

### 3.3. Table 3 Regression Results of Adult Children’s Statuses on Parents’ Depressive Symptoms

Table 3 shows the OLS regression results of adult children’s individual status attainments and associations with their parents’ depressive symptoms. Each column corresponds to an adult status, including educational attainment, romantic relationship status, residence, employment status, parenthood, arrest, and all status attainments included (Models 1–7). We conducted stepwise regression models, first without controls, and then including controls for each adult status.

Overall, adult children’s educational attainment and romantic relationship status were positively associated with depressive symptoms among their parents, providing partial support for Hypothesis 1, that parents of children who experience the acquisition of transitions to adulthood associated with greater resources will have better overall health. Shown in Model 1, parents whose adult children completed some college, a college degree, and more than a college degree had significantly fewer depressive symptoms (0.20-point, 0.22-point, and 0.19-point fewer depressive symptoms, respectively) relative to adult children who did not complete a high school degree. Including controls, parents whose children completed a college degree or more had significantly fewer depressive symptoms (0.22-point and 0.19-point fewer depressive symptoms, respectively), relative to adult children who did not complete a college degree.

In additional to educational attainment, the romantic relationship status of the adult child was significantly associated with parents’ depressive symptoms (Model 2). Relative to parents of adult children who were married, parents of adult children who were never married had a 0.10-point increase in depressive symptoms. This relationship also held true once all transitions to adulthood and controls were included; relative to parents whose adult children were married, parents whose adult children were never married reported a 0.08-point increase in depressive symptoms. Parents of adult children who experienced a separation, divorce, or widowhood also had an increase in depressive symptoms, though this association was not statistically significant. This finding provides support for Hypothesis 2, that parents whose adult children experience transitions to adulthood that are associated with fewer resources, such as never being married, experience worse mental health.

Although the adult child’s employment status was not significantly associated with parents’ depressive symptoms (Model 4), other transitions to adulthood that were significantly associated with increased depressive symptoms included the adult child’s residence and whether the adult child had ever been arrested (Models 3 and 5). Parents of adult children who were living in another arrangement, such as group quarters, had 0.16-points higher depressive symptoms, but this association was no longer significant with the addition of controls. Parents of adult children who had ever been arrested had 0.07-point higher depressive symptoms in the basic model. However, once controls were included, this association similarly no longer remained significant.

With all adult status attainments included (Model 7), the statuses of adult children significantly associated with increased depressive symptoms among parents only included adult children’s educational attainment. With all controls included, parents of adult children who had completed a college degree had 0.20-point fewer depressive symptoms relative to parents of adult children who completed less than a high school degree. Although not central to the study, factors that were significantly associated with depressive symptoms among parents included the parents’ gender, the age of the parent, and parental education. Women and older parents were significantly less likely than men and younger parents to have increased depressive symptoms. Parents who completed a college degree or more had significantly fewer depressive symptoms relative to parents who completed less than a high school degree. In sum, we found evidence in support of Hypothesis 1, that the attainment of certain transitions to adulthood associated with greater resources was linked to improved mental health among parents, and Hypothesis 2, that adult status attainments that were associated with fewer resources tied to poorer mental health for parents.

**Table 3:** OLS regressions predicting depressive symptoms of parents based on transitions to adulthood experienced by their adult children.

	Model 1		Model 2		Model 3		Model 4		Model 5		Model 6		Model 7	
	Ed. Attainment		Relationship		Residence		Employment		Parenthood		Ever Arrested		All Statuses	
Educational attainment of adult child—High School or General Education Development (rel. to <High School)	−0.12	−0.10											−0.11	−0.10
	(0.09)	(0.09)											(0.09)	(0.09)
Some college	−0.20 *	−0.16											−0.18 *	−0.14
	(0.08)	(0.08)											(0.08)	(0.08)
Completed college	−0.29 ***	−0.22 **											−0.27 **	−0.20 *
	(0.08)	(0.08)											(0.09)	(0.09)
More than college	−0.29 ***	−0.19 *											−0.27 **	−0.17
	(0.08)	(0.09)											(0.09)	(0.09)
Relationship status of adult child—Separated/divorced/widowed (rel. to married)			0.06	0.05									0.04	0.03
			(0.03)	(0.03)									(0.04)	(0.04)
Never married			0.10 **	0.08 *									0.07	0.06
			(0.03)	(0.03)									(0.04)	(0.04)
Living arrangement of adult child—Live with parents (rel. to living alone)					0.01	−0.02							−0.08	−0.07
					(0.05)	(0.05)							(0.05)	(0.05)
Other living arrangement					0.16 *	0.14							0.08	0.09
					(0.08)	(0.08)							(0.09)	(0.08)
Employment status of adult child—Employed (rel. to unemployed)							−0.03	0.01					0.01	0.02
							(0.04)	(0.04)					(0.04)	(0.04)
Parenthood status of adult child—Ever had child (rel. to never had a child)									−0.03	−0.04			−0.02	−0.03
									(0.03)	(0.03)			(0.03)	(0.03)
Ever arrested (rel. to never arrested)											0.07 *	0.06	0.02	0.03
											(0.03)	(0.03)	(0.03)	(0.03)
Gender of parent—Woman		0.15 ***		0.14 **		0.15 **		0.15 ***		0.15 ***		0.15 ***		0.14 **
		(0.04)		(0.04)		(0.05)		(0.04)		(0.04)		(0.05)		(0.05)
Race of parent—Black (rel. to white)		0.07		0.05		0.07		0.07		0.07		0.07		0.05
		(0.05)		(0.05)		(0.05)		(0.05)		(0.05)		(0.05)		(0.05)
Latinx		−0.03		−0.03		−0.02		−0.03		−0.03		−0.03		−0.02
		(0.05)		(0.05)		(0.05)		(0.05)		(0.05)		(0.05)		(0.05)
Asian		−0.01		−0.02		−0.02		−0.01		−0.02		−0.02		−0.02
		(0.08)		(0.08)		(0.08)		(0.08)		(0.08)		(0.08)		(0.08)
Age of parent at Wave V		0.02		0.01		0.01		0.02		0.01		0.01		0.02 *
		(0.01)		(0.01)		(0.01)		(0.01)		(0.01)		(0.01)		(0.01)
Immigrant generation status of parent—Born outside of the U.S.		0.07		0.07		0.06		0.06		0.06		0.07		0.08
		(0.08)		(0.08)		(0.08)		(0.08)		(0.08)		(0.08)		(0.08)
Parental education—High School or General Education Development (rel. to <High School)		−0.07		−0.08		−0.09		−0.09		−0.09		−0.08		−0.08
		(0.06)		(0.06)		(0.06)		(0.06)		(0.06)		(0.06)		(0.06)
Some college		−0.08		−0.11 *		−0.11 *		−0.11 *		−0.11 *		−0.11 *		−0.09
		(0.06)		(0.06)		(0.06)		(0.06)		(0.06)		(0.06)		(0.06)
College		−0.14 *		−0.18 **		−0.19 **		−0.18 **		−0.19 **		−0.18 **		−0.15 **
		(0.06)		(0.06)		(0.06)		(0.06)		(0.06)		(0.06)		(0.06)
College and more		−0.24 ***		−0.29 ***		−0.30 ***		−0.30 ***		−0.30 ***		−0.30 ***		−0.26 ***
		(0.06)		(0.05)		(0.05)		(0.05)		(0.05)		(0.05)		(0.06)
Constant	1.63 ***	1.54 ***	1.37 ***	1.47 ***	1.40 ***	1.50 ***	1.43 ***	1.50 ***	1.43 ***	1.56 ***	1.39 ***	1.45 ***	1.59 ***	1.53 ***
	(0.08)	(0.21)	(0.02)	(0.20)	(0.01)	(0.21)	(0.03)	(0.20)	(0.02)	(0.21)	(0.01)	(0.21)	(0.10)	(0.23)
N	1548													

Standard errors in parentheses. * *p* < 0.05; ** *p* < 0.01; *** *p* < 0.001.

### 3.4. Table 4 Regression Results of Adult Children’s Statuses and Parents’ ADL Conditions

Table 4 shows the OLS regression results of adult children’s statuses and parents’ summed ADL conditions. Similar to Table 3, each column corresponds to an adult status, which includes educational attainment, romantic relationship status, residence, employment status, parenthood, arrest, and all statuses included in Models 1–7. We conducted stepwise regression models for each adult status attainment, first without controls, and then with controls.

Overall, transitions to adulthood that were significantly associated with decreased ADL conditions among midlife parents included the educational attainment of the adult child, the romantic relationship status of the adult child, and the employment status of the adult child. Greater educational attainment among adult children was associated with decreased ADL conditions among parents (Model 1). In the basic model, parents of adult children who completed some college degree, completed a college degree, and completed more than a college degree had approximately 0.78, 1.32, and 1.38 fewer ADL conditions relative to parents whose adult children had completed less than a high school degree. With the addition of controls, however, parents of adult children who completed college and had completed more than a college degree had significantly fewer ADL conditions (1.06 fewer conditions). Therefore, this provides evidence to support Hypothesis 1, that parents of adult children who experience status attainments associated with greater resources will have better physical health.

Similar to educational attainment, the romantic relationship status of the adult child was also associated with parents’ ADL conditions. In the basic model (Model 2), relative to parents of adult children who were married, parents of adult children who had experienced a separation, divorce, or widowhood, or were never married, had experienced an increase in ADL conditions of 0.40 points and 0.41 points, respectively. Including controls, however, only parents of adult children who had never been married had a significant increase in ADL conditions by 0.36 points relative to parents of adult children who were married. Therefore, there is evidence to support Hypothesis 2, that adult status attainments associated with fewer resources among adult children are linked to worse parental physical health. In addition, the adult child’s employment status—specifically, whether or not they were employed—was also significantly associated with parents’ ADL conditions. In both the basic model and the model with controls, parents of adult children who were employed had a significant decrease in ADL conditions by 0.99 points in the model without controls, and 0.78 points in the model including controls, thus providing support for Hypothesis 1.

With all adult status attainments and controls included, the educational attainment of the adult child, the romantic relationship status of the adult child, and the employment status of the adult child all remained significantly associated with ADL conditions. In particular, greater educational attainment, marriage, and employment were associated with decreased ADL conditions among parents. Other controls that were significantly associated with decreased ADL conditions included if the parent was younger in age, and if the parent had completed at least some college degree or more. Therefore, we found evidence to support Hypothesis 1, that adult status attainments that were linked to greater resources would be positively associated with parental physical health, and Hypothesis 2, that adult status attainments that were linked to fewer resources would be negatively associated with parental physical health.

**Table 4:** OLS regressions predicting parental sum of ADL conditions based on adult status changes experienced by adult children.

		Model 1		Model 2		Model 3		Model 4		Model 5		Model 6		Model 7	
		Ed. Attainment		Relationship		Residence		Employment		Parenthood		Ever Arrested		Transitions to Adulthood	
Educational attainment of adult child—High school or GED (rel. to <High School)		−0.63	−0.58											−0.58	−0.54
		(0.35)	(0.35)											(0.35)	(0.36)
Some college		−0.78 *	−0.63											−0.69 *	−0.56
		(0.33)	(0.33)											(0.33)	(0.33)
Completed college		−1.32 ***	−1.06 **											−1.16 ***	−0.93 *
		(0.34)	(0.35)											(0.35)	(0.36)
More than college		−1.38 ***	−1.06 **											−1.17 ***	−0.88 *
		(0.35)	(0.36)											(0.35)	(0.37)
Relationship status of adult child—Separated/divorced/widowed (rel. to married)				0.40 *	0.36									0.35	0.34
				(0.18)	(0.19)									(0.19)	(0.19)
Never married				0.41 **	0.36 *									0.42 *	0.38 *
				(0.15)	(0.16)									(0.17)	(0.18)
Living arrangement of adult child—Live with parents (rel. to living alone)						0.41	0.31							−0.04	−0.06
						(0.23)	(0.23)							(0.26)	(0.25)
Other living arrangement						0.27	0.25							−0.22	−0.15
						(0.30)	(0.30)							(0.30)	(0.31)
Employment status of adult child—Employed (rel. to unemployed)								−0.99 ***	−0.78 **					−0.57 ***	−0.56 **
								(0.25)	(0.27)					(0.17)	(0.17)
Parenthood status of adult child—Ever had child (rel. to never had a child)										0.16	0.12			0.25	0.23
										(0.14)	(0.14)			(0.16)	(0.16)
Ever arrested (rel. to never arrested)												0.22	0.21	0.00	0.06
												(0.14)	(0.14)	(0.15)	(0.15)
Gender of parent—Woman		0.20		0.16		0.20			0.17		0.18		0.19		0.15
		(0.38)		(0.37)		(0.38)			(0.39)		(0.37)		(0.37)		(0.39)
Race of parent—Black (rel. to white)		0.30		0.23		0.31			0.34		0.34		0.31		0.22
		(0.20)		(0.21)		(0.20)			(0.20)		(0.20)		(0.20)		(0.21)
Latinx		0.24		0.23		0.22			0.26		0.25		0.24		0.22
		(0.27)		(0.28)		(0.27)			(0.28)		(0.28)		(0.28)		(0.28)
Asian		−0.44		−0.40		−0.39			−0.41		−0.38		−0.44		−0.43
		(0.53)		(0.53)		(0.53)			(0.52)		(0.53)		(0.53)		(0.52)
Age of parent at Wave V		0.02		0.01		0.01			0.02		0.01		0.01		0.02 *
		(0.01)		(0.01)		(0.01)			(0.01)		(0.01)		(0.01)		(0.01)
Immigrant generation status of parent—Born outside of the U.S.		−0.04		−0.09		−0.13			−0.10		−0.12		−0.07		0.01
		(0.32)		(0.33)		(0.33)			(0.33)		(0.33)		(0.33)		(0.32)
Parental education—High School or GED (rel. to <High School)		−0.39		−0.48 *		−0.49 *			−0.47		−0.48 *		−0.49 *		−0.39
		(0.25)		(0.25)		(0.24)			(0.24)		(0.24)		(0.24)		(0.25)
Some college		−0.57 *		−0.75 **		−0.75 **			−0.69 **		−0.73 **		−0.75 **		−0.54 *
		(0.25)		(0.25)		(0.25)			(0.25)		(0.25)		(0.25)		(0.25)
College		−0.84 **		−1.08 ***		−1.06 ***			−1.00 ***		−1.05 ***		−1.07 ***		−0.82 **
		(0.29)		(0.28)		(0.28)			(0.28)		(0.28)		(0.28)		(0.30)
College and more		−1.18 ***		−1.48 ***		−1.50 ***			−1.43 ***		−1.50 ***		−1.51 ***		−1.14 ***
		(0.33)		(0.31)		(0.31)			(0.31)		(0.31)		(0.31)		(0.33)
Constant	−0.23	−1.50	−1.33 ***	−1.77	−1.21 ***	−1.67		−0.58 ***	−1.34	−1.27 ***	−1.70	−1.23 ***	−1.77	−0.20	−1.73
	(0.31)	(1.26)	(0.09)	(1.20)	(0.07)	(1.22)		(0.15)	(1.23)	(0.12)	(1.21)	(0.08)	(1.22)	(0.37)	(1.30)
N	1548														

Standard errors in parentheses. * *p* < 0.05; ** *p* < 0.01; *** *p* < 0.001.

## 4. Discussion

In the period of the delayed transition to adulthood, the length of time in which parents and children have overlapping needs and responsibilities to take care of each other has increased [4,51]. We explored the degree to which children’s transitions to adulthood were associated with parents’ depressive symptoms and ADL conditions, using nationally representative, longitudinal data from the National Study of Adolescent to Adult Health and the Add Health Parent Study. We explored a variety of adult status attainments, including educational attainment, romantic relationships, residence, employment, parenthood, and incarceration, and their associations with parental health. In sum, our results demonstrated the importance of adult children’s status attainments on midlife parents’ mental and physical health. Specifically, we found that parents whose adult children completed a college degree or more had fewer depressive symptoms relative to parents whose adult children completed less than a high school degree. We also found that parents whose adult children attained higher levels of education, were married, and were employed had fewer ADL conditions relative to parents whose adult children had less than a high school degree, were never married, and were unemployed. Therefore, we found support for the life course perspective (Elder, 1994)—parents are invested in and influenced by their children’s life circumstances, supporting the concept of linked lives. In addition, we found support for the stress contagion theory [4]—stressors that young adults face may also influence their parents’ mental and physical health. The results of this paper support two key themes: that certain adult status attainments, specifically educational attainment, shape parents’ mental health, and that adult status attainments, including educational attainment, marriage, and employment, shape parents’ physical health.

The first theme that emerged from this study was that adult status transitions, specifically educational attainment, shape parents’ mental health. When examining multiple types of status transitions, the only one that was significantly associated with parents’ decreased depressive symptoms was the adult child’s higher educational attainment, including controls and all other adult status attainments. Although the period of time in which young adults spend pursuing higher education has increased over the past few decades [9], thus potentially increasing the amount of time parents provide support to their children, higher educational attainment increases young adults’ capability to provide for themselves through labor market opportunities, greater lifetime earnings, access to social capital, and may even be a source of parental pride [30,52]. In turn, this may decrease worry and stress among parents, in that it alleviates the burden of parents to provide additional financial, instrumental, and emotional resources to their children [4,32]. In addition, children who have completed a college degree may be more equipped to provide additional financial resources for their parents in return, thus reducing strain and potentially improving mental health for parents [29,32]. This finding confirms other studies that have found that adult children’s college completion is linked to decreased depressive symptoms among parents [30,31,32].

The second theme that emerged from this study was that status transitions shape parents’ physical health. Specifically, educational attainment, marriage, and employment among adult children were all associated with decreased ADL conditions among parents. These specific transitions to adulthood are linked to increased financial resources among adult children [34,52,53], which in turn may relate to parental health through various mechanisms: First, by decreasing worry and strain among parents to provide for their children financially, parents may have better physical health. Second, children with greater educational attainment and financial resources may be better able to transmit health-promoting information related to physical health [29,31]. Third, children with greater educational attainment and financial resources may have more flexible careers that allow them more opportunities to take care of parents with functional limitations [29]. Our findings confirm previous studies that have found links to adult children’s higher educational attainment and improved parental physical health and longevity [29,31,54,55]. We contribute to the literature by revealing that parents of adult children who are married or employed have fewer ADL conditions relative to parents of adult children who have never been married or are unemployed. This finding is also reflected in prior studies that have found marriage and employment status to be linked to improved health among individuals [56,57].

Despite these findings, our results revealed that certain markers of adulthood—such as changes in residence, entry into parenthood, and incarceration—were not associated with parents’ health. There might be several explanations for these findings. Indeed, a move back to the parental home could indicate adult children’s increased reliance on their parents [39,40]. However, co-residence may allow the adult child new opportunities to provide parents with instrumental and emotional support, thereby benefitting the parents’ health [58]. Likewise, moving out could signal fewer instrumental or emotional support exchanges flowing from adult children to parents. The relationship between a child’s entry into parenthood and the midlife parents’ health may be contingent on other factors, such as the adult child’s age at first birth or their partnership status [33,44]. As for incarceration, this was measured by asking if the child was ever arrested. Therefore, the relationship between a child’s incarceration and the parents’ health could be influenced by several other factors, including the offense for which the child was arrested, the duration of incarceration, and how recently the arrest occurred.

This study was not free from limitations. Although these findings inform how children’s status attainment relates to parental health, there are several future directions for this study that can increase our understanding of this relationship. Currently, we examine how individual markers of adulthood for children are linked to parental health. However, the acquisition of one status often does not happen in a vacuum. Children often experience much variability on the road to adulthood, which may involve multiple changes in status during a given period of time [59]. Future research can examine how changes in adult children’s transitions to adulthood during a given timeframe may shape parental health, for example, entry and exit from marriage. In addition, we did not conduct a causal analysis, so our results may be affected by omitted variable bias in that parental factors may predict adult children’s ability to complete transitions to adulthood, which may in turn shape parental health. In addition, we do not examine the mechanisms through which status attainments shape parental health, only the status attainment itself. For example, parents’ increased stress or anxiety may be a mechanism linking adult children’s status attainments to parents’ health, which is an avenue for future research. Finally, this study was focused on the United States, where there are fewer social welfare policies that support young adults and midlife parents compared to other welfare states [60]. Thus, future research can examine whether these findings may differ if this study were replicated using data from a country with a stronger social welfare system. Despite these limitations, there were several strengths to this study, including the use of dyadic, nationally representative data, the examination of several transitions to adulthood, and rich measures on parental health outcomes.

## 5. Conclusions

To conclude, our study reveals that children’s educational attainment was linked to fewer activities of daily living (ADL) limitations and depressive symptoms among parents. Children’s marriage and employment were also associated with fewer ADL limitations among parents. Taken together, our findings illustrate how the mental physical health of parents can be shaped by their adult children’s status achievements. Several interpretations can be derived from the central findings of this study. With growing economic precarity and declining population health due to COVID-19, many adult children face greater financial hardships, moving back into the parental home, and difficulty securing full-time employment [61]. We suspect adult children’s hardships with securing transitions to adulthood will not only be linked to worsened mental and physical health among adults themselves, but may also have spillover consequences for their parents. Our work also lends itself to support policies that provide greater economic stability among young adults—promoting and making the attainment of a college degree more affordable, for example—which can improve not only adults’ own well-being, but also promote their parents’ mental and physical health. Investing in young adults’ ability to complete status attainments can have positive mental and physical health implications for older generations [29,62].

## Data Availability

The authors used the restricted use Add Health and Add Health Parent Study datasets. Information on access to Add Health and the Add Health Parent Study is available here: https://addhealth.cpc.unc.edu/data/ (accessed on 2 March 2023).
